# A comparison of transient experiential wellbeing across health enhancing behaviours in the American Time Use Survey

**DOI:** 10.1038/s41598-026-40985-7

**Published:** 2026-02-24

**Authors:** Jessica K. Bone, Feifei Bu, Jill K. Sonke, Daisy Fancourt

**Affiliations:** 1 Research Department of Behavioural Science and Health, Institute of Epidemiology & Health Care, University College London, 1-19 Torrington Place, London, WC1E 7HB UK; 2 Center for Arts in Medicine, University of Florida, Gainesville, USA

**Keywords:** Health care, Psychology, Psychology

## Abstract

Health-enhancing behaviours can support long-term subjective wellbeing. However, less research has examined experiential wellbeing (how people feel in the moment). By comparing experiential wellbeing during different health-enhancing behaviours, we could learn which dimensions of behaviours promote health. We included 11,144 participants from the American Time Use Survey who engaged in health-enhancing behaviours and reported levels of positive (happiness) and negative (sadness, stress, tiredness, pain) affect. Multilevel linear regression models tested how affect differed during eight behaviours. Social engagement was most common, followed by reading and physical activity. Attending sports events was least common. Pain and tiredness were higher during physical activity than other behaviours. Happiness was highest when doing receptive arts (attending performing arts, museums, watching dance, listening to music) and lowest when volunteering and reading. Results for sadness and stress were more variable. Activities that were done with others, outside the home, and that were very meaningful were associated with more positive and less negative affect. Overall, health-enhancing behaviours evoked complex emotional reactions, with positive and negative affect experienced simultaneously. Recommendations around health-enhancing leisure activities in Western countries focus on physical activity, but our findings support calls to include more activities alongside guidance on the context in which people engage.

**Supplementary Information:**

The online version contains supplementary material available at 10.1038/s41598-026-40985-7

## Introduction

Health behaviours include any activity done, or abstained from, to improve health or wellbeing and/or to prevent or detect illness^1^. They are generally divided into three categories: health-enhancing behaviours, which provide health benefits or protect against ill health; health-impairing behaviours, which harm health or increase risk of disease; and behaviours for detecting health problems^2^. Although most research on health-enhancing behaviours has focussed on a specific set of behaviours (e.g. exercise, diet, socialising), other leisure activities can support health, so could also be considered health-enhancing behaviours. Defined as voluntary non-work activities that are participated in for pleasure^3^, leisure includes everyday activities such as hobbies, arts, culture, sports, volunteering, and community groups. Engagement in hobbies has been associated with lower levels of depression, higher life satisfaction, and better perceived health across countries^4^. Similarly, arts and cultural activities can be used to prevent, manage, and treat illness^5,6^. Volunteering and religious activities have also been related to outcomes from mental health to mortality^7–10^.

Wellbeing is frequently studied in relation to health-enhancing behaviours. Yet, wellbeing is a multidimensional umbrella term, with varying definitions across disciplines. One common model is to distinguish eudaimonic from hedonic wellbeing^11–15^. Eudaimonic wellbeing relates to finding meaning and flourishing (e.g. mastery, autonomy, personal growth) but is not the focus of this study. Hedonic wellbeing, in contrast, focusses on attaining pleasure and avoiding pain and can be further divided into evaluative wellbeing and experiential wellbeing. Evaluative wellbeing includes judgements about one’s current situation in relation to one’s own beliefs and goals (e.g. life satisfaction). Experiential wellbeing comprises how people feel in the moment, with day-to-day emotions ranging from joy to misery to pain, and can be measured through ratings of these momentary positive and negative affective states^11–15^. To date, there has been more research on the effects of health-enhancing behaviours on evaluative than experiential wellbeing^16–20^. Yet evaluative and experiential wellbeing are weakly related^12,18,21,22^, differentially patterned across socioeconomic groups^18,23^, and independently related to health outcomes^23,24^. Understanding how health-enhancing behaviours influence experiential wellbeing is thus as important as evaluative wellbeing. Additionally, health-enhancing behaviours are complex and multidimensional^25^, each including a range of active ingredients that may improve health (e.g. movement, social interactions, cognitive demands)^26^. By directly comparing experiential wellbeing during health-enhancing behaviours, we may learn which attributes of different activities are most important for health.

The gold standard for measuring experiential wellbeing is ecological momentary assessment (EMA), whereby people report levels of positive and negative affect following prompts throughout the day^27,28^. Using EMA with 200 older adults, positive affect was highest during social and spiritual behaviours^29^. Another study using EMA with 67 older adults found that socialising and cultural activities had the highest levels of happiness^30^. However, EMA is demanding, limiting studies to small non-representative samples who can respond regularly^25^. One alternative is the day reconstruction method (DRM)^31^, in which people recall all activities done on a specific day and rate their affect during the activities. The DRM can be used more easily with larger samples and approximates the real-time reports made during EMA^31^. The DRM has thus been incorporated into some time use surveys, allowing for measurement of mood during activities in a specific period. For example, in the American Time Use Survey (ATUS), individuals report all the activities they engaged in over the last 24 h. A supplementary Wellbeing Module then measured how people felt during selected activities^32^. This involved using the DRM for three randomly selected activities from the last day, with individuals reporting how happy, sad, stressed, tired, and in pain they felt during each selected activity. We refer to this as a partial DRM, as it was only completed for a subset of activities from the previous day.

Two analyses of ATUS have used the partial DRM to compare average daily affect among those who did and did not engage in specific health-enhancing behaviours on the survey day. In these nationally representative samples of adults in the United States (US; *n* = 8,746, *n* = 33,924), those who did voluntary activities reported higher levels of happiness^33,34^. In a multi-country study that also used a partial DRM with 21,000 older adults, spending time socialising was associated with lower levels of negative affect than being alone^35^. But the challenge with comparing average daily affect is that findings might be driven by differences in other activities throughout the day. It would instead be better to compare affect ratings during specific activities. Another study using the ATUS partial DRM (*n* = 26,289) has shown that people experience greater happiness during leisure activities than work or domestic chores, with happiness during voluntary and religious activities also high^23^. Separating out different leisure activities, physical activity was associated with greater happiness and less sadness than passive leisure (screen time, relaxing) in 422 matched older adults from ATUS^36^. However, no research has yet compared experiential wellbeing across many different health-enhancing behaviours simultaneously.

A further consideration is that health-enhancing behaviours may occur in different contexts, and this could alter their influence on wellbeing. Activities that happen in social contexts or include social interactions may be particularly important, as social activities that meet our affiliative needs can have greater impacts on wellbeing^13^. In line with this, another analysis of ATUS DRM data found that happiness during non-work activities was consistently lower when these activities were performed alone^37^. In a smaller study using EMA with 200 older adults, all activities were enjoyed more if a friend was present^29^. Where activities are done is also relevant: another EMA study of 67 older adults found that active leisure was most strongly related to happiness when done outdoors^30^. In a survey of 7272 English adults, those who spent time in nature reported greater happiness^38^. Similarly, peoples’ experiences are likely influenced by how meaningful a behaviour is to them^13^. Health-enhancing behaviours differ in meaning, with physical activity often rated as more meaningful than passive leisure^36^. In 21,736 ATUS participants, activities rated as more meaningful were linked to higher levels of positive affect in the DRM, although it was less clear whether they were related to lower negative affect^27^. Given that behaviours vary in social context, location, and meaningfulness, exploring whether these factors influence experiential wellbeing will help us to understand the underlying mechanisms responsible for improving health and indicate effective health promotion strategies.

To date, it remains unclear whether and how (a) experiential wellbeing varies across health-enhancing behaviours and (b) the context of health-enhancing behaviours alters their relationship with wellbeing. Therefore, in this study, we focussed on eight health-enhancing behaviours with established long-term health benefits, namely physical activity, sports events, participatory arts, receptive arts, reading, social engagement, religious/spiritual activities, and volunteering. Our first objective was to test whether these activities were differentially associated with experiential wellbeing. We measured experiential wellbeing using ratings of positive affect (happiness) and negative affect (sadness, stress, tiredness, pain), collected using the partial DRM in ATUS. Our second objective was to test whether associations with affect differed according to the social context, location, and meaningfulness of each activity. We hypothesized that there would be different patterns of affect across activities, with positive affect highest for social engagement, volunteering, and arts activities. We also expected activities done with others, outside the home, and more meaningful activities to be associated with more positive and less negative affect.

## Methods

### Sample

**Fig. 1:**
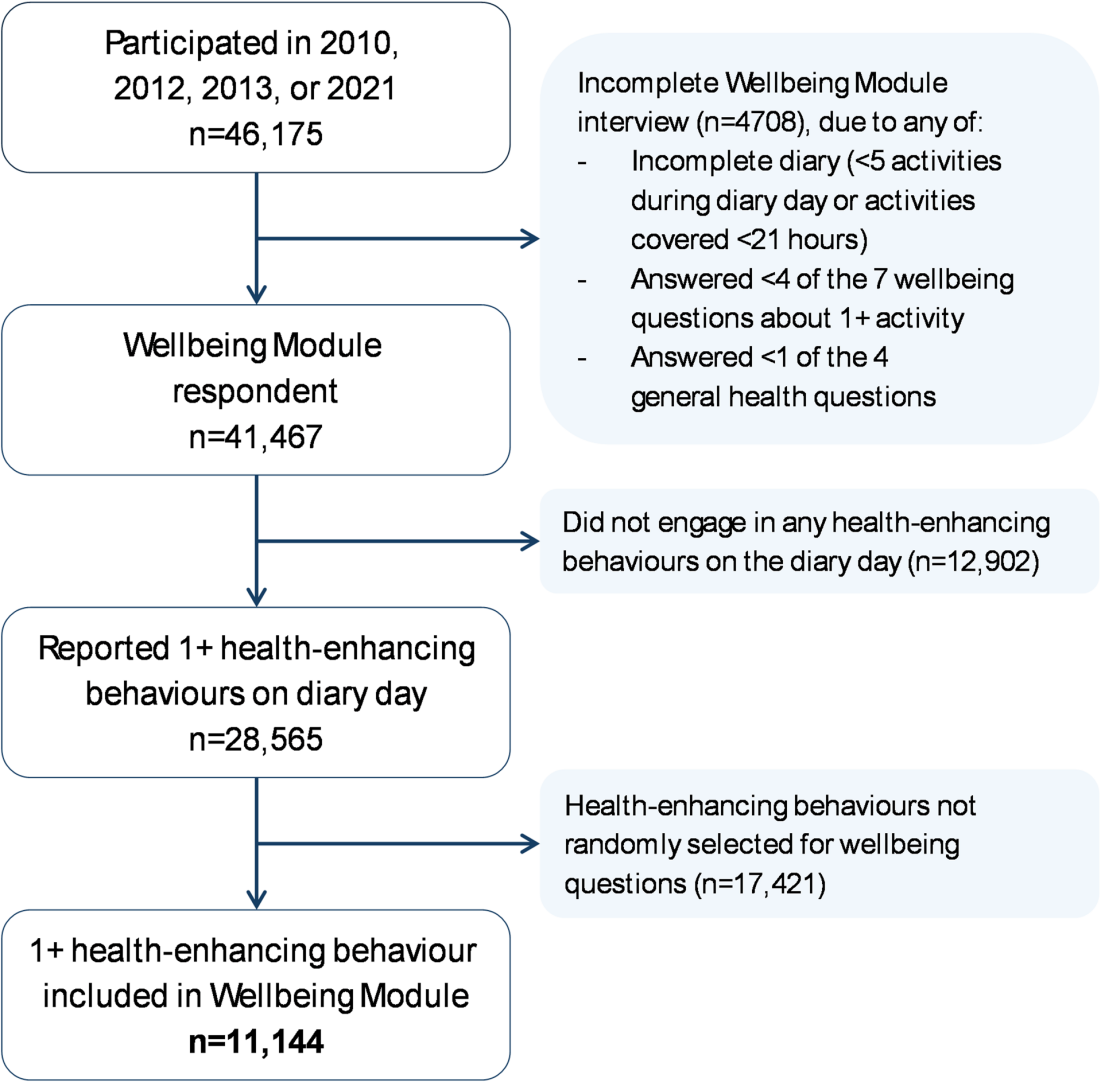
Flowchart showing sample selection, from overall ATUS sample to analytical sample.

ATUS is a continuous cross-sectional survey, with interviews conducted nearly every day from 2003 onwards by US Census Bureau. ATUS is representative of all residents of private households in the US aged 15 and over^32^. Participation is evenly distributed across the year. We combined and analysed data from the ATUS years in which a Wellbeing Module was fielded (2010, 2012, 2013, 2021). ATUS response rates in these years were 56.9%, 53.2%, 49.9%, and 39.4% respectively^32^. For all participants, three activities from the last day were randomly selected for the Wellbeing Module. ATUS included 41,467 participants in the Wellbeing Module dataset across years (Fig. 1). We further limited our sample to participants who (a) reported engaging in one or more health-enhancing behaviours on the diary day (*n* = 28,565) and (b) had one or more of these health-enhancing behaviours randomly selected for the Wellbeing Module. This left a final analytical sample of 11,144 participants (12,923 observations).

### Ethical approval

This analysis has approval from University of Florida Institutional Review Board (IRB202401080) and University College London Research Ethics Committee (project 18839/001). All participants gave informed consent, and all research was performed in accordance with the Declaration of Helsinki.

### Measures

Participants recalled their activities over 24 h, beginning at 4am on the day prior to the interview and ending at 4am on the day of the interview. Participants were randomly assigned a day of the week on which to complete the survey, with 10% allocated to each weekday and 25% to each weekend day. Weights account for this non-uniform distribution and differing response rates, providing estimates for an average day. Participants reported every primary activity during the 24-hour period. Activities were coded using a standard lexicon, verified by two coders.

In the Wellbeing Module, three activities from the last day were randomly selected (lasting ≥ 5 min, excluding sleeping/grooming/personal activities). From 1st January 2010 to 24th March 2013, there was an error in the activity selection process, resulting in the systematic exclusion of the last eligible activity in participants’ diaries in most cases. Consequently, eligible activities that occurred at or near the end of the diary period, often a period of TV watching, were underrepresented. ATUS activity weights account for this error.

#### Health-enhancing behaviours

We included all health-enhancing behaviours measured in ATUS, each consisting of a range of specific activities. There were eight types of behaviours: physical activity; sports events; participatory arts; receptive arts; reading; social engagement; religious/spiritual activities; volunteering (Table 1; full details in Table S1). Participatory arts included actively creating or making art (e.g. arts and crafts, performing, writing, dancing, extracurricular music and performance), whereas receptive arts included consumption of art that has been created and is now experienced by an audience (e.g. attending performing arts, museums, watching dance, listening to/playing music). Listen to/playing music was included in receptive arts because we expected the receptive forms of this activity (listening to recorded music, listening to others make music) to be more common than actively producing music, and it was more strongly related to other receptive than participatory arts activities. Behaviours included only instances where these were individuals’ main activities, as information on secondary activities was not collected (e.g. where music was on in the background whilst doing other activities).

**Table 1:** Engagement rate and time spent engaging for each health-enhancing behaviour.

Behaviour	Included activities	Engagement rate	Minutes engaged
Physical activity	Aerobics; biking; running; golfing; walking; working out; etc	20%	137.83 (6.56)
Sports events	Watching boating; watching football; watching soccer; watching vehicle touring/racing; watching wrestling; etc.	1%	191.55 (17.52)
Participatory arts	Extracurricular music and performance activities; performing; arts and crafts as a hobby; writing for personal interest; dancing; arts and crafts with (non-)household children	3%	180.05 (18.67)
Receptive arts	Listening to/playing music (not radio); attending performing arts; attending museums; watching dancing	3%	148.22 (10.18)
Reading	Reading for personal interest; reading to or with (non-) household children	20%	111.64 (6.01)
Social engagement	Socialising and communicating with others; attending or hosting parties/receptions/ ceremonies; attending meetings for personal interest (not volunteering); telephone calls to or from family members/ friends/neighbours/ acquaintances	47%	161.60 (3.88)
Religious/spiritual activities	Religious television; attending religious services; participation in religious practices; religious education activities	9%	131.33 (5.40)
Volunteering	Volunteer administrative and support activities; volunteer social service and care activities (except medical); volunteer indoor and outdoor maintenance, building, and clean-up; volunteer attending meetings conferences and training; volunteer public health activities; volunteer public safety activities	4%	152.66 (11.07)

For full details of the specific activities within each behaviour type, see Table S1.

Note. *n* = 11,144 participants (12,923 observations). Minutes engaged calculated only for those who engaged (participation mean), with mean (standard error) shown in the table. Weighted results from 40 multiply imputed datasets.

#### Social context

For each health-enhancing behaviour, we created a binary indicator of whether participants were interacting with anyone during the activity, including over the phone (yes, no). Online interactions were not specifically mentioned, so may not have been counted.

#### Location

We used a binary indicator of whether each health-enhancing behaviour was done at home (participant’s or another person’s home) or outside the home (e.g. workplace, library, school, outdoors, place of worship).

#### Meaning

In the Wellbeing Module, participants rated how meaningful each activity was to them on a seven-point scale, from not at all to very meaningful. As most health-enhancing behaviours were perceived as meaningful, we dichotomised these ratings to indicate activities that were less meaningful (rated 0–5) versus activities that were very meaningful (rated 6).

#### Wellbeing

Participants completed five affect ratings, reporting how happy, sad, stressed, tired, and in pain they felt during each selected activity on a seven-point scale from not at all to very. Pain was rated from not feeling any pain at all to being in severe pain, but type of pain was not specified. We measured affect for each health-enhancing behaviour by pooling the scores for specific activities within those categories (e.g. all kinds of physical activity). In most cases, this involved taking the rating for a specific activity as an indicator for the overarching behaviour as most participants reported only a single activity in each category. Where participants reported on multiple activities within the same category, ratings were averaged.

#### Individual characteristics

We measured a range of demographic, socioeconomic, and health-related factors that have been associated with health-enhancing behaviours and wellbeing^2^. Data were collected during the ATUS interview or from Current Population Survey (CPS) interviews 2–5 months earlier (mean = 3.00, standard deviation [SD] = 0.56). Demographic factors included sex (male, female), age (years), race (White, Black, Asian, Other [American Indian/Alaskan Native/Hawaiian/Pacific Islander/Mixed race]), ethnicity (non-Hispanic, Hispanic), marital status (married, widowed/divorced/separated, never married), household size, whether there was a child under 18 in the household, metropolitan area (non-metropolitan area, metropolitan area), and region in which the participant lived (Northeast, Midwest, South, West).

Socioeconomic factors included education (high school or less, college, undergraduate, postgraduate), employment status (employed, unemployed, not in labour force, retired), annual family income (quartiles: less than $30,000, $30,000 - $59,999, $60,000 - $99,999, $150,000 and over), and whether the participant provided eldercare on the diary day.

Health-related factors included self-rated health (poor, fair, good, very good, excellent) and whether participants had a disability that prevents work, high blood pressure, felt well-rested on the diary day (not at all, a little, somewhat, very), and took pain medication on the diary day. Participants also rated life satisfaction on the Cantril ladder from the worst possible (0) to the best possible life (10) and reported their overall feelings yesterday, compared to the same day of the week typically (better, the same, worse).

### Statistical analysis

First, descriptive statistics explored sample characteristics, engagement in health-enhancing behaviours, and levels of affect during an average minute spent in each behaviour. Next, multilevel linear regression models tested whether affect differed across the eight health-enhancing behaviours. This accounted for the clustering of activities within individuals, as people might interpret the scales differently, meaning within-individual measures were not independent^31^. Given that most participants were only eligible to report affect for one behaviour, we modelled within- and between-individual variation. We included a random intercept, allowing it to vary across participants. Models are reported after adjustment for confounders, with unadjusted results in Table S4. Confounders included all individual characteristics, meaningfulness, social context, and location. We additionally adjusted for activity duration, time spent in all eligible activities, time since CPS completion, day of the week, and survey year.

We used two ATUS weights in analyses. In descriptive analyses, individual-level weights accounted for complex sampling, day of the week, and response rates, enabling estimates for an average day representative of the US civilian non-institutionalised population aged 15 and over. In the main analyses, activity-level weights additionally accounted for the time respondents spent doing each activity, the total time spent in eligible activities, and the probability that a specific activity was sampled. These weights address the issue that participants who did activities more often were more likely to be represented. As we do not know how frequently participants did each activity, we cannot estimate average affect for the population. Instead, weighting allows us to estimate affect for an average minute the population spends in the activity.

Missingness was low (≤ 8%; Table S3), except for life satisfaction and overall feelings yesterday, which were introduced in 2012 (31% missing). ATUS replaced missing income data using previous CPS waves for 15%. Multiple imputation by chained equations accounted for data that were still missing^39^. We generated 40 imputed datasets using linear, truncated linear, logistic, and ordered logistic regression according to variable type, with all variables used in analyses, sampling weights, and interactions for effect modifiers included. Findings did not differ to complete case analyses (Table S8), so imputed results are reported.

#### Effect modifiers

We explored the proportion of participants for each health-enhancing behaviour who (a) interacted with others, (b) engaged outside the home, and (c) found it meaningful. We then examined whether the associations between health-enhancing behaviours and affect differed according to these characteristics. To do this, we repeated the main analysis, adding an interaction term between behaviour type and each effect modifier in separate models.

#### Sensitivity analyses

The main analyses used physical activity as the reference category, as this behaviour has most consistently been associated with positive health outcomes. However, in supplementary analyses, we changed the reference category to (a) social engagement, to compare to spending time with others, and (b) reading, as the most passive health-enhancing behaviour. Additionally, we used an index of net affect, calculated as the happiness rating minus the sadness rating for each activity, which indicated how participants were feeling overall during each activity^31^.

## Results

Overall, 41,467 individuals responded to the ATUS Wellbeing Module, reporting on an average of 2.97 activities (range 1–3). Of these, 11,144 engaged in a health-enhancing behaviour and had this activity randomly selected for the Wellbeing Module. These participants had a mean age of 47 (SD = 20), and 53% identified as female, 51% were married, 54% employed, 13% Hispanic, 81% White, 12% Black, 5% Asian, and 2% identified as American Indian, Alaskan Native, Hawaiian/Pacific Islander, or multiple racial groups (Table 2). Overall, 85% of participants lived in metropolitan areas and 5% had a disability that prevented work. The characteristics of our analytical sample were similar to the overall sample (Table S2).

**Table 2:** Descriptives statistics for the analytical sample (*n* = 11,144).

Characteristic	Category	Mean (SD)
Age		46.99 (20.08)
Household size		2.91 (1.58)
Life satisfaction		7.18 (1.87)
		Proportion
Gender	Female	53%
Race	White	81%
	Black	12%
	Asian	5%
	Other	2%
Ethnicity	Hispanic	13%
Marital status	Married	51%
	Widowed/divorced/separated	17%
	Never married	31%
Child under 18 in household	Yes	35%
Metropolitan area	Metropolitan	85%
Region	Northeast	18%
	Midwest	23%
	Central	36%
	West	23%
Education	High school or less	43%
	College	23%
	Undergraduate	21%
	Postgraduate	13%
Employment status	Employed	54%
	Unemployed	6%
	Not in labour force	19%
	Retired	22%
Annual family income	Less than $30,000	25%
	$30,000 - $59,999	27%
	$60,000 - $99,999	24%
	$100,000 and over	25%
Provided eldercare	Yes	4%
Self-rated health	Excellent	19%
	Very good	35%
	Good	30%
	Fair	12%
	Poor	4%
Disability that prevents work	Yes	5%
High blood pressure	Yes	31%
Well-rested	Very	43%
	Somewhat	38%
	A little	14%
	Not at all	5%
Took pain medication	Yes	28%
Feelings typical yesterday	Better	29%
	The same	61%
	Worse	10%
Survey year	2010	25%
	2012	26%
	2013	26%
	2021	23%

Note. Weighted results from 40 multiply imputed datasets.

Each participant reported on 1–2 health-enhancing behaviours (mean = 1.16). Social engagement was most common (engaged in by 47% of participants; Table 1), followed by reading (20%), and physical activity (20%). Attending sports events was least common (1%). The average time spent on each health-enhancing behaviour ranged from 1 h to 52 min (reading) to 3 h and 12 min (sports events).

### Health-enhancing behaviours and affect

**Fig. 2:**
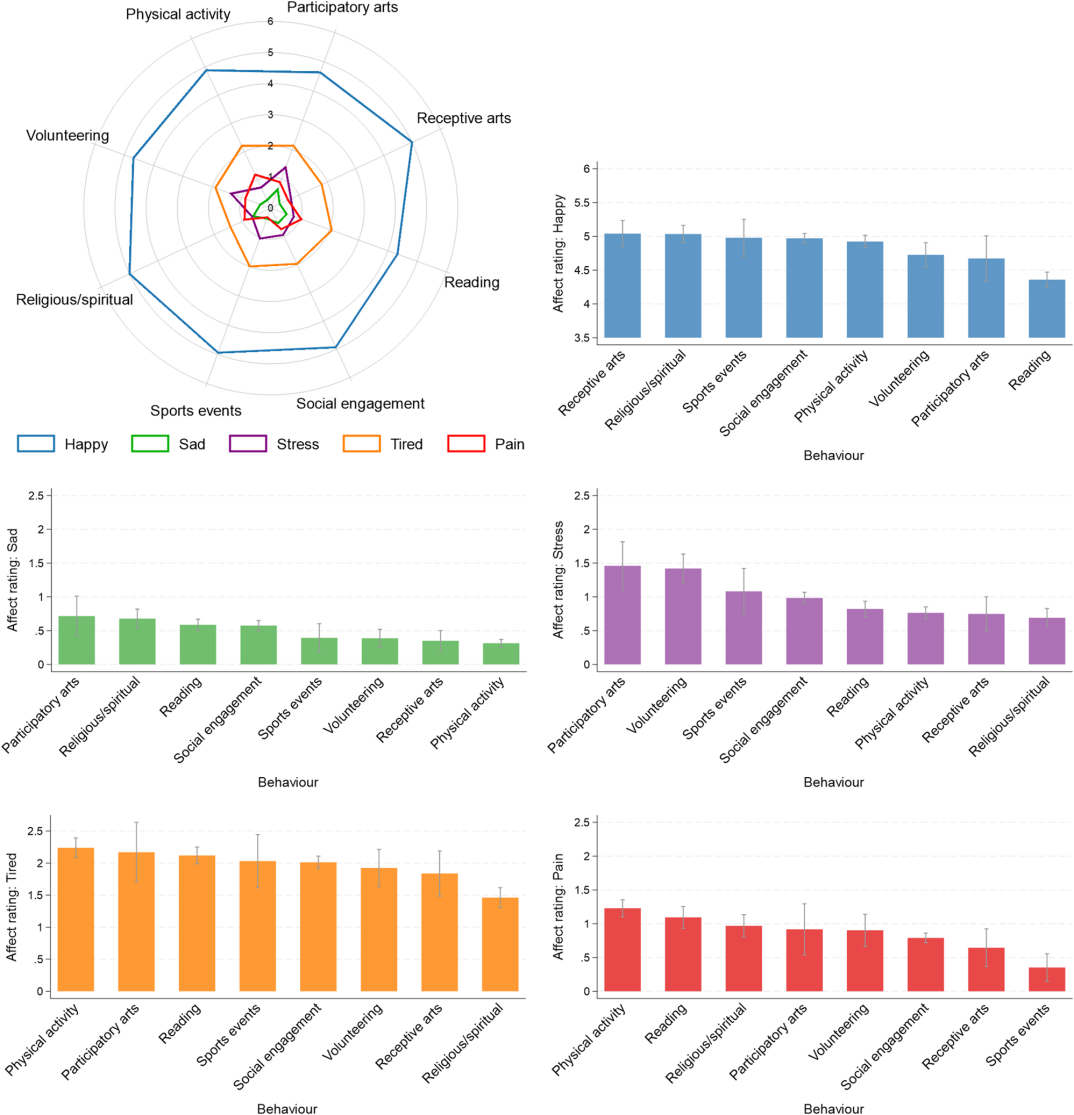
Descriptive statistics, showing mean affect ratings for an average minute spent in each health-enhancing behaviour, weighted and from 40 multiply imputed datasets. Bar charts also show 95% confidence intervals.

Descriptively, levels of affect for an average minute spent in each behaviour varied across behaviours (Fig. 2). Happiness was highest when doing receptive arts activities and lowest when reading. Sadness was lowest for physical activity, and highest, alongside stress, for participatory arts. Stress and tiredness were lowest for religious/spiritual activities. Tiredness and pain were highest for physical activity. Finally, pain was lowest when attending sports events.

**Fig. 3:**
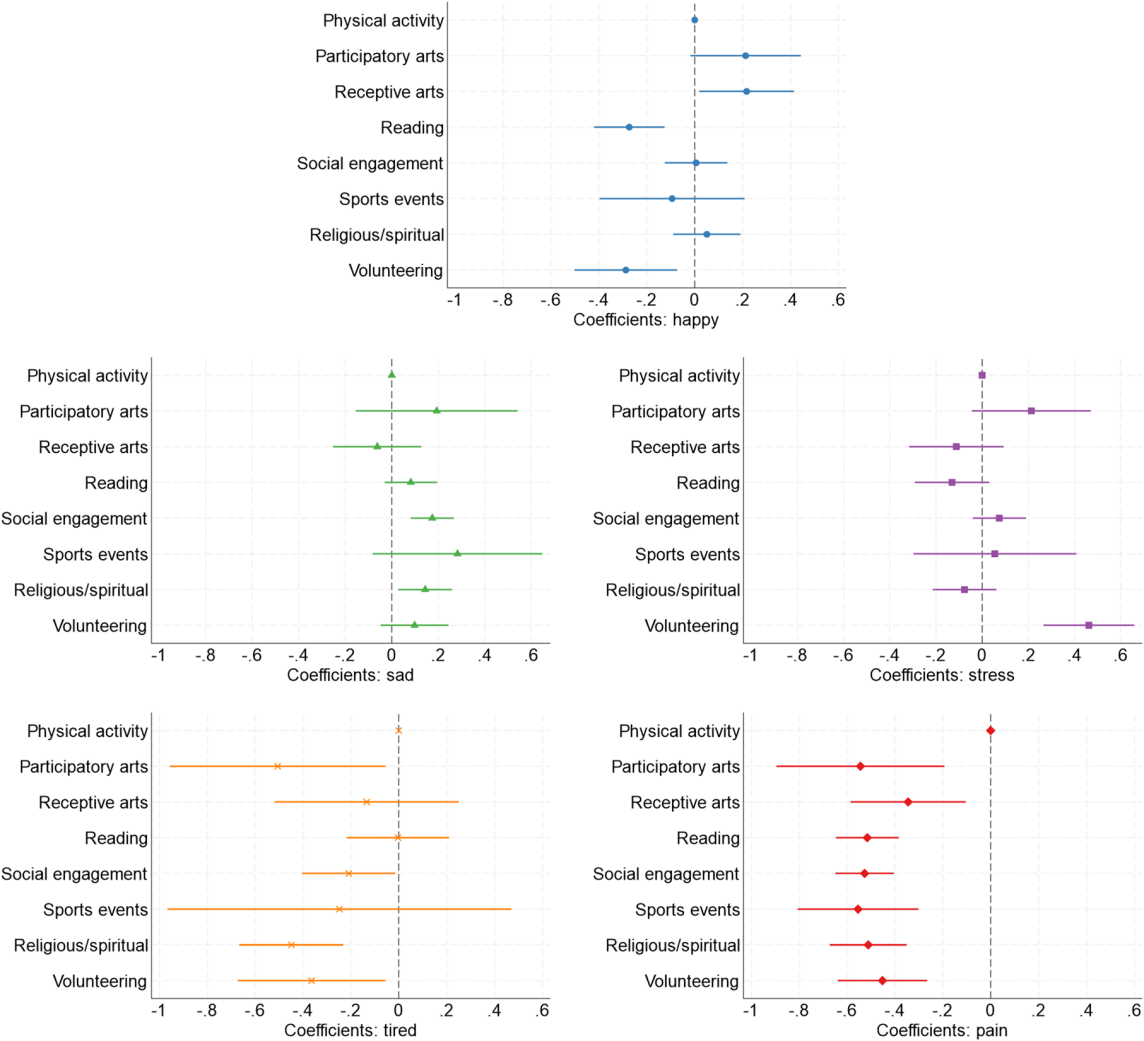
Coefficients and 95% confidence intervals from multilevel linear regression models testing whether affect differed across the eight health-enhancing behaviours, adjusted for confounders, weighted and from 40 multiply imputed datasets. Coefficients represent the difference in affect rating, compared to physical activity, for an average minute spent in each health-enhancing behaviour.

In adjusted multilevel linear regression models, compared to doing physical activity, happiness was higher when doing receptive arts but lower when volunteering or reading (Fig. 3). In contrast, sadness trended towards being higher in most activities (except for receptive arts), although there was only evidence for differences in social engagement and religious/spiritual activities versus physical activity. Stress showed less consistent patterns, with evidence that stress was higher only when volunteering than during physical activity. Tiredness was generally lower amongst all activities than physical activity, with evidence for lower tiredness during religious/spiritual activities, volunteering, participatory arts, and social engagement. Finally, pain was consistently rated lower for all other behaviours than physical activity, with the largest differences for participatory arts and sports events.

### Effect modifiers

#### Social context

**Fig. 4:**
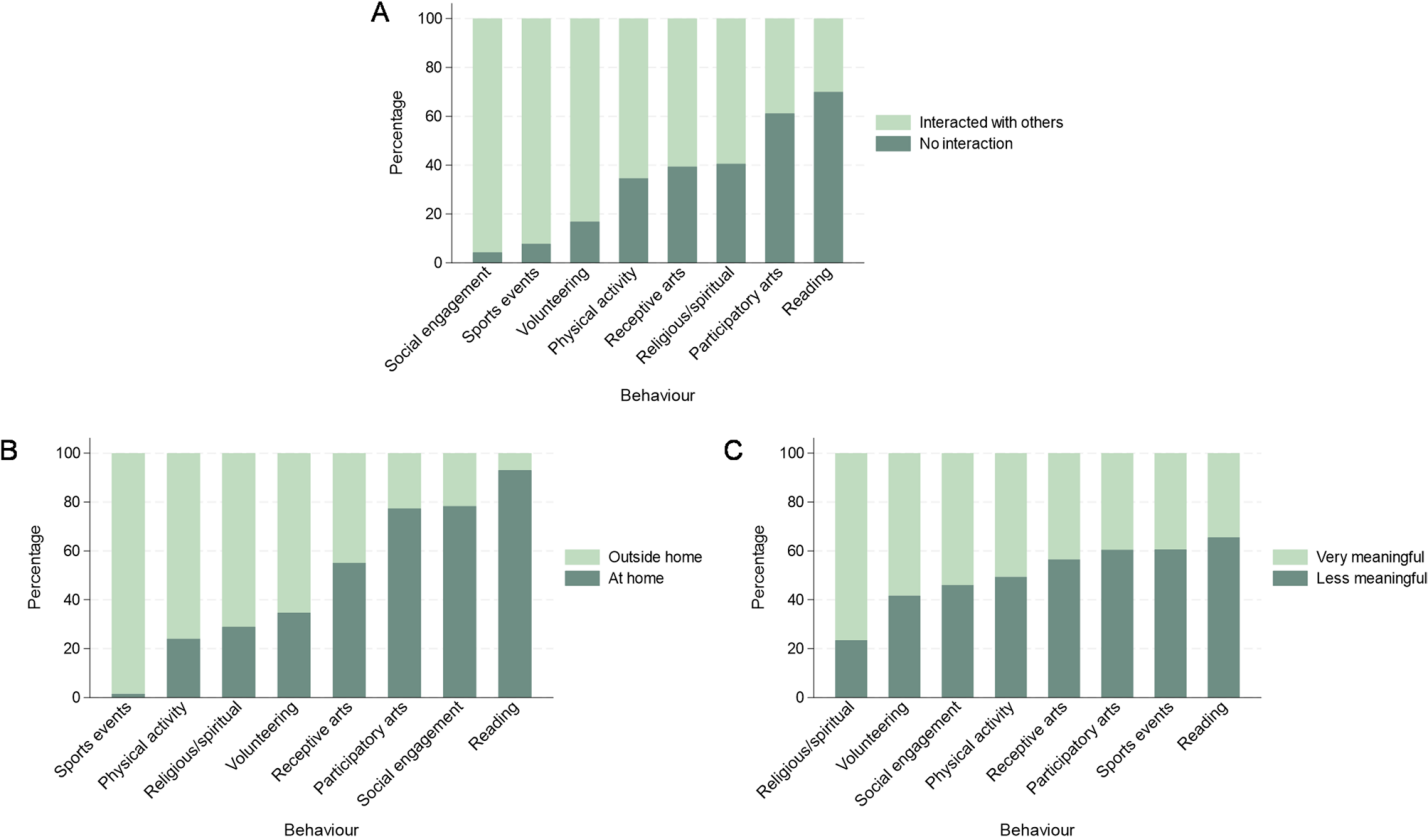
Descriptive statistics showing the proportion of each health-enhancing behaviour that (A) involved interaction with others, (B) took place outside the home, and (C) was meaningful, weighted and from 40 multiply imputed datasets.

Social engagement most often involved interaction with others (96% of activities), followed by sports events (92%), and volunteering (83%; Fig. 4A). Participants interacted with others least when reading (30%). When health-enhancing behaviours were done with others, happiness was consistently higher and sadness, stress, and pain were generally lower than when engaging alone. However, most associations with affect did not differ by social context. There was only weak evidence that the relationship with happiness for participatory arts versus physical activity varied (Table S5). When not interacting with others, happiness was higher for participatory arts than physical activity, but this difference was not present when interacting with others (Fig. S1).

#### Location

Sports events were mostly attended outside the home (98% of activities), followed by physical activity (76%), and religious/spiritual activities (71%; Fig. 4B). Reading was mainly done at home (93%). Health-enhancing behaviours done outside the home were generally linked with higher happiness, stress, and tiredness but lower sadness and pain than those done at home. There was some evidence that associations between behaviours and happiness, stress, tiredness, and pain differed according to location (Table S6). However, patterns of effect modification varied across behaviours and outcomes. The most consistent difference was for volunteering; when at home, volunteering was associated with lower happiness, more stress, and similar pain as physical activity (Fig. S2). When outside home, there were smaller differences in happiness and stress, and lower pain for volunteering versus physical activity.

#### Meaning

Most activities were meaningful, with religious/spiritual activities most often rated as very meaningful (76%), followed by volunteering (58%), social engagement (54%), and physical activity (51%; Fig. 4C). Reading was rated as very meaningful least often (34%). Across all behaviours, happiness was higher and all four types of negative affect lower when activities were very meaningful (versus less meaningful). Associations with stress were most likely to differ by meaning, although there was also a consistent difference for religious/spiritual activities across happiness, stress, and tiredness (Table S7). When activities were less meaningful, happiness was lower, stress higher, and tiredness was similar for religious/spiritual activities versus physical activity. When very meaningful, there was no difference in happiness, and stress and tiredness were lower for religious/spiritual activities (Fig. S3). There was also a similar pattern in stress for social engagement and volunteering; people experienced more stress compared to physical activity only when activities were less meaningful.

### Sensitivity analyses

Using social engagement as the reference category (Fig. S4), happiness was only higher during receptive arts and was lower when volunteering and reading. Sadness was lower during receptive arts and physical activity. Stress was higher when volunteering, but lower when reading and doing religious/spiritual activities. Tiredness was higher when reading and doing physical activity, but lower in religious/spiritual activities than social engagement. Pain ratings were similar across all behaviours except physical activity, for which people reported more pain.

Taking reading as the reference category instead (Fig. S5), participants reported greater happiness during most other behaviours. There were no differences for sadness, and again pain was only higher for physical activity. Stress was higher during volunteering, participatory arts, and social engagement than when reading. Finally, tiredness trended towards being lower among all other behaviours, except physical activity.

Finally, looking at net affect (Fig. S6), this was lower for reading and volunteering than physical activity, suggesting that the difference between happiness and sadness was smaller for reading and volunteering than for physical activity.

## Discussion

Among over 11,000 US adults, social engagement was the most common health-enhancing behaviour, followed by reading and physical activity. Attending sports events was least common. When people attended sports events, they spent the most time doing so (over three hours). People spent two to three hours per day on all health-enhancing behaviours on average. Looking at experiential wellbeing, the most consistent associations were for pain and tiredness, which were higher in physical activity than other health-enhancing behaviours. Happiness trended towards being highest when doing receptive arts and lowest when doing volunteering and reading. Results for sadness and stress were more variable, with stress showing the greatest moderation depending on context.

Our findings demonstrate that health-enhancing behaviours can evoke complex emotional reactions, with positive and negative affect experienced simultaneously. During physical activity, people report moderate happiness, low sadness and stress, but high tiredness and pain. Elevated tiredness and pain during physical activity is consistent with previous research^40^ and likely relates to the challenging nature of many activities with longer-term benefits for physical and mental health^29,41^. Additionally, feeling tired and experiencing muscle soreness during physical activity is often considered a positive benefit of exercise (as a result of successful exertion that can improve strength and fitness), so it might not represent negative affect in the same way as other activities. Yet physical activity may not be unique in providing a combination of positive and negative affect. People also reported moderate happiness and high sadness, stress, and tiredness during participatory arts. This has been called the “tragedy paradox”: despite trying to minimise negative affect in everyday life, people actively seek out experiences of negative emotions in the arts, potentially as a way of feeling moved without the consequences of a real sad or distressing event^42,43^. This may be therapeutic, providing opportunities to experience emotional connection, build predictive mental models for emotional experiences, and practice emotion regulation^44^.

In contrast to participatory arts, receptive arts were more consistently linked to enhanced experiential wellbeing, with the highest happiness and low negative affect across all domains. Religious/spiritual activities were also associated with high positive and lower negative affect. Given that receptive arts and religious activities are related to long-term health and mortality, transient experiential wellbeing could form “micropatterns” that have a mechanistic role in long-term outcomes^4–10^. Both behaviours provide opportunities for experiencing awe and contemplation^26^, are associated with moving ‘peak’ experiences^45^, and often involve aesthetically pleasing spaces. Receptive arts have even been linked to feelings of spirituality and proposed as a form a secular worship^46^. Many religious and spiritual activities also include elements of the arts, such as singing, choirs, chanting, poetry, and visual art.

It was surprising that volunteering and social engagement had average levels of affect, as previous studies have suggested that they are among the most enjoyable activities^29,30,33–35^. This shows the utility of directly comparing health-enhancing behaviours, although it is important to acknowledge that differences could be explained by ATUS definitions. ‘Volunteering’ included organisational and administrative tasks that may be less rewarding than volunteering in people-facing roles^47^. Similarly, social engagement included hosting parties/receptions/ceremonies, arguing with family/friends, and attending meetings for personal interest, which may be less enjoyable than casual face-to-face socialising^48^. Future research should consider more specific combinations of ingredients within volunteering and social engagement to explore if there are key components that contribute to experiential wellbeing^11^.

Health-enhancing behaviours varied in social context, location, and meaning. Religious/spiritual activities were done with others two thirds of the time, mostly outside the home, and were most frequently rated as very meaningful. Volunteering was next highest in meaning and often involved interacting with others outside the home. When done at home or when less meaningful, people reported more negative affect for volunteering than physical activity, but these differences were smaller or reversed when volunteering was outside the home or very meaningful. Similarly, negative affect was only lower for religious/spiritual activities and social engagement when these behaviours were very meaningful. Volunteering, religious/spiritual activities, and socialising may thus enhance wellbeing only when they are very meaningful or outside the home. This is concerning given that people increasingly spend their leisure time at home^37^. There was little evidence that other associations differed according to social context, location, and meaningfulness. This does not mean that these factors are unimportant, however. Positive affect was generally higher and negative affect lower when activities were done with others, outside the home, and were very meaningful. This could also explain why reading was generally associated with low positive and high negative affect, as it was most often done alone, at home, and less meaningful.

This study has several strengths. We included over 11,000 people, with weights enabling us to estimate the level of affect for an average minute the population spends in each behaviour. Although response rates declined over time, ATUS measured all daily activities, so there should be less selection bias than surveys explicitly focussed on specific behaviours. Time use surveys are less susceptible to recall bias than other surveys that require reporting over longer periods. The DRM corresponds well to EMA, with good test-retest reliability^49^. Although ATUS used a partial DRM, with only three activities selected per person, these were chosen randomly. However, the partial DRM has limitations. Less than half of the relevant activities were included, and we had to limit our sample accordingly. Of the 41,467 ATUS Wellbeing Module respondents, 69% (*n* = 28,565) engaged in one or more health-enhancing behaviours on the diary day. However, only 39% (*n* = 11,144) of these respondents had a health-enhancing behaviour randomly selected for the partial DRM, so were included in our study. This resulted in little within-individual variation, because few participants reported wellbeing for more than one health-enhancing behaviour. As specific activities were uncommon on the diary day, and rarely included in the Wellbeing Module, we had to group them into the eight behaviours. Future research should aim to replicate our findings in time use surveys using a full DRM to enable assessment of engagement rates for different health-enhancing behaviours, exploration of each behaviour in more detail, and study of within-individual variation in affect.

Furthermore, analyses of effect modification may have been underpowered, as some events were rarely done in certain contexts (e.g. sports events alone/at home). Social context focussed on interacting with others, including over the phone. This may mean activities done in the presence of others but not actively interacting with them, or involving virtual interactions, were incorrectly classified as being done alone. This could explain why only 96% of social engagement involved interactions. Additionally, 85% of participants were living in metropolitan areas, meaning the sample lacked representation of rural residents. Finally, ATUS involved retrospective reports, meaning we could not test the direction of relationships. Behaviour was likely influenced by current mood^14^, as well as subsequently changing mood. People could have been more likely to do receptive arts or religious/spiritual activities when they were happy, and less likely to do physical activity when they were sad. People often reported feeling tired, sad, and in pain whilst reading, which could be due to reading before sleeping, or when they did not have the energy for more active behaviours. It is difficult to tease out the direction of these relationships, as experiential wellbeing constantly fluctuates^15,31^. However, testing whether associations are influenced by time of day might help to reveal the factors underlying differences in experiential wellbeing (e.g. people are likely to feel more tired late in the day), so this is recommended in future research.

Although there is ongoing debate about how they are related, experiential wellbeing is likely to influence longer-term evaluative wellbeing^13,21,22,27^ as well as subsequent behavioural choices^18^ and health outcomes^23,24^. Focusing on eight health-enhancing behaviours with established long-term health benefits, we found differential associations with experiential wellbeing across activities, likely attributable to different active ingredients involved. This suggests that it is important to engage in a range of health-enhancing behaviours for maximal benefits. Activities that are done with others, outside the home, and are very meaningful may be particularly beneficial. Currently, recommendations around health-enhancing leisure activities in Western countries focus on physical activity^2,50^, but our findings support calls for recommendations to include a wider range of activities and guidance on the context in which people engage.

## Supplementary Information

Below is the link to the electronic supplementary material.

Supplementary Material 1

## Data Availability

This study used publicly available ATUS data, available from: [https://www.bls.gov/tus/data.htm](https:/www.bls.gov/tus/data.htm).
