# Supplementary material for: A comparison of transient experiential wellbeing across health enhancing behaviours in the American Time Use Survey
Source: Sci Rep. 2026 Feb 24;16:10410. doi: 10.1038/s41598-026-40985-7 (PMC13031343; doi:10.1038/s41598-026-40985-7)
Supplement: Supplementary file 1 — Supplementary Material 1 [file 41598_2026_40985_MOESM1_ESM.docx]

**Supplementary Materials**

| **Contents** | **Page** |
| --- | --- |
| Table S1. Health-enhancing behaviours measured in ATUS. | 2 |
| Table S2. Characteristics of wellbeing module sample. | 6 |
| Table S3. Missingness in study variables. | 8 |
| Table S4. Unadjusted main analysis. | 9 |
| Table S5. Effect modification by social context. | 9 |
| Table S6. Effect modification by location. | 10 |
| Table S7. Effect modification by meaningfulness. | 10 |
| Table S8. Main analysis limited to complete cases. | 11 |
| Figure S1. Stratified by social context. | 12 |
| Figure S2. Stratified by location. | 13 |
| Figure S3. Stratified by meaningfulness. | 14 |
| Figure S4. Social engagement as reference category. | 15 |
| Figure S5. Reading as reference category. | 15 |
| Analysis of net affect. | 16 |
| Figure S6. Main analyses using net affect. | 16 |

# **Table S1.** Health-enhancing behaviours measured in ATUS, with the examples provided in 2021.

| **Behaviour** | **ATUS activity categories** | **Examples (where provided)** |
| --- | --- | --- |
| Physical activity | Doing aerobics | doing step aerobics, low-impact aerobics, high-impact aerobics, Zumba, talking to aerobics instructor |
|  | Playing baseball | talking to baseball coach |
|  | Playing basketball | talking to basketball referee |
|  | Biking | registering for a bike race, spinning, riding a stationary bike, biking/mountain biking |
|  | Playing billiards | shooting pool, playing snooker |
|  | Boating | sailing, yachting, kayaking, rafting, canoeing, crewing |
|  | Bowling | playing duck pins, playing ten pins |
|  | Climbing, spelunking, caving | rock climbing, mountain climbing |
|  | Participating in equestrian sports | horseback riding, playing polo, steeple chase |
|  | Fencing |  |
|  | Fishing | deep-sea fishing, ice fishing, fly fishing, crabbing, clam-digging, talking to the boat captain |
|  | Playing football | playing touch football, playing flag football, talking to the football referee |
|  | Golfing | playing the front 9, playing the back 9, practicing putting, talking to the caddy |
|  | Doing gymnastics | tumbling, using the balance beam, using parallel bars, using uneven bars, using the pommel horse, talking to the gymnastics coach |
|  | Hiking |  |
|  | Playing hockey | playing ice hockey, playing field hockey, talking to the hockey coach |
|  | Hunting | hunting game, hunting deer/elk, hunting ducks, talking to the hunting guide |
|  | Participating in martial arts | doing karate, doing kickboxing, doing tae kwon do, talking to martial arts instructor |
|  | Playing racquet sports | playing tennis, squash, handball, racquetball, badminton, lacrosse, talking to the tennis coach |
|  | Participating in rodeo competitions | calf roping, barrel riding |
|  | Rollerblading | roller-skating, inline skating, skateboarding |
|  | Playing rugby |  |
|  | Running | jogging, running a marathon, running a race/organized run, running cross country, running on a treadmill, talking to race officials |
|  | Skiing, ice skating, snowboarding | snow tubing, sledding |
|  | Playing soccer |  |
|  | Softball | playing wiffle ball |
|  | Using cardiovascular equipment | using the Stairmaster, elliptical, rowing machine, treadmill, exercising on the treadmill |
|  | Vehicle touring/racing | stock car racing, drag racing, doing motocross, snowmobiling, 4-wheeling, riding in a go-cart, riding in or driving a dune buggy |
|  | Playing volleyball | playing Wally ball |
|  | Walking | walking on a treadmill, power walking, speed walking |
|  | Participating in water sports | swimming, playing water polo, diving, waterskiing, snorkeling, scuba diving, river tubing, surfing, body surfing, boogie-boarding, talking to the swimming coach, water aerobics |
|  | Weightlifting/strength training | lifting weights, bodybuilding |
|  | Working out, unspecified | stretching, doing calisthenics, doing cardio (unspecified), doing Crossfit |
|  | Wrestling | freestyle wrestling, talking to the wrestling referee, Greco-Roman wrestling |
|  | Doing yoga | Pilates, talking to the yoga instructor |
|  | Playing sports not elsewhere classified | bungee jumping, cricket, tai chi, paintball, baton twirling, curling, cheerleading, footbag, setting up camping tent, starting a campfire, digging a latrine, collecting firewood for camp, playing in the snow, ballooning, skydiving, windsurfing, kite flying, parkour, ping-pong, table tennis, horseshoes, croquet, archery, skeet-shooting, target practice, throwing darts, boxing, shooting league, javelin, pole vaulting, discus, playing Frisbee golf, throwing a Frisbee, hang-gliding, playing ultimate frisbee, attending track practice |

| Receptive arts | Listening to/playing music (not radio) | listening to recorded music/records/CDs/DVDs/tapes, playing musical instrument (leisure), singing/singing karaoke/Christmas caroling, listening to someone play the piano, composing music, tuning musical instruments |
| --- | --- | --- |
|  | Attending performing arts | attending comedy club, opera, a concert, the ballet, dance troupe performances, plays, musicals, the theater, jazz bar |
|  | Attending museums | attending an art gallery, visiting the zoo, attending exhibitions, attending an arboretum, attending a botanical garden |
|  | Watching dancing |  |
| Participa-tory arts | Extracurricular music & performance activities | attending band practice, attending choir practice as extracurricular school activity, attending play practice |
|  | Performing | acting in a performance (volunteer), dancing in a performance (volunteer), performing music (volunteer), emceeing a charity function (volunteer), auctioneering a benefit auction (volunteer), practicing for a performance (volunteer) |
|  | Arts and crafts as a hobby | scrapbooking, making a scrapbook, making holiday/other decorations, videotaping/photography/model making/jewelry making, making pottery/sculpting/wood working, taking pictures/snapshots/photographs, making a TikTok video, making Halloween costumes (for self), dyeing Easter eggs, artistic painting, making Christmas decorations, sketching/drawing, making podcast |
|  | Writing for personal interest | writing in diary/journal (personal interest), writing lyrics, blogging (personal interest), writing stories (personal interest), editing (personal interest) |
|  | Dancing |  |
|  | Arts and crafts with hh children | making holiday decorations w/hh child, helping hh child make arts and crafts, organizing coin collection with hh child, building model planes with hh child |
|  | Arts and crafts with nonhh children | making holiday decorations w/nonhh child, helping nonhh child make arts and crafts, organizing coin collection with nonhh child, building model planes with nonhh child |
| Reading | Reading for personal interest | reading a magazine/book/newspaper (personal interest), flipping/leafing through magazine (personal interest), listening to books on tape/audio books (personal interest), borrowing books from the library, doing research (personal interest), checking out library books, being read to (personal interest), reading, unspecified, returning library books/browsing at the library, reading a book on a Kindle or other electronic book reader (personal interest) |
|  | Reading to/with hh children | reading to or with hh child, listening to a hh child read, helping hh child read, teaching hh child to read |
|  | Reading to/with nonhh children | reading to or with nonhh children, listening to a nonhh child read, helping nonhh child read, teaching nonhh child to read |
| Social engage-ment | Socializing and communicating with others | entertaining family, hanging out with family, spending time with family, talking with family, arguing with family, greeting family, hugging or kissing family, visiting with family, accompanying family while they run errands, entertaining friends, hanging out with friends, spending time with friends, talking with friends, arguing with friends, greeting friends, hugging/kissing friends, visiting with friends, hugging/kissing acquaintances/others, greeting other parents, hugging/kissing neighbors, giving gifts to friends, giving presents to adults, opening birthday presents (with others), opening Christmas gifts (with others), opening gifts (with others), opening wedding presents (with others), visiting household/nonhh adult in hospital, visiting adult in nursing home, accompanying family/friends to look at family/friend's new house, talking with neighbors, talking to/with other parents, talking with acquaintances, greeting neighbors/acquaintances, accompanying friends while they run errands |
|  | Attending or hosting parties/receptions/ceremonies | attending graduation ceremonies (for adults), attending a charity social event, attending receptions, attending parties, attending birthday parties, attending bachelor/ette party, attending housewarming, attending holiday open house |
|  | Attending meetings for personal interest (not volunteering) | attending a senior citizens meeting, attending an Al-anon meeting, attending a homeowner's association meeting, attending a fraternal lodge meeting, attending AA meetings, attending a Weight Watchers meeting, attending a Boy Scout/Girl Scout meeting (for self), attending professional meetings (not work), attending fraternity/sorority meetings, attending book club meetings, attending club meetings (not school) |
|  | Telephone calls to/from family members | talking on phone to relatives, placing a call to my mother on Amazon Echo or similar technology, Skyping with my sister, FaceTime/Video chat with family |
|  | Telephone calls to/from friends, neighbors, or acquaintances | talking on phone to friends/neighbors/acquaintances, talking on phone to boyfriend, texting with a friend, talking on phone to girlfriend, talking on phone to fiancee, FaceTime/Video chat with friends |

| Sports events | Watching aerobics |  |
| --- | --- | --- |
|  | Watching baseball |  |
|  | Watching basketball |  |
|  | Watching biking | watching a bicycle race |
|  | Watching billiards | watching pool players, snooker players |
|  | Watching boating | watching sailing, kayaking, canoeing, yachting, rafting, crewing |
|  | Watching bowling | watching ten pins, duck pins |
|  | Watching climbing, spelunking, caving |  |
|  | Watching equestrian sports | watching horseback riding, a steeple chase, polo game |
|  | Watching fencing |  |
|  | Watching fishing | watching deep-sea fishing, fly fishing, crabbing, clam-digging |
|  | Watching football | watching touch football, tag football |
|  | Watching golfing | watching a golf tournament |
|  | Watching gymnastics | watching tumbling, balance beam completion, parallel bar competition |
|  | Watching hockey | watching ice hockey, field hockey |
|  | Watching martial arts | watching karate, kickboxing |
|  | Watching racquet sports | watching tennis, squash, handball, racquetball, badminton, lacrosse |
|  | Watching rodeo competitions |  |
|  | Watching rollerblading | watching roller-skating, skateboarding |
|  | Watching rugby |  |
|  | Watching running | watching a marathon, a running race, cross country running, an organized run |
|  | Watching skiing, ice skating, snowboarding | watching snow tubing |
|  | Watching soccer |  |
|  | Watching softball | watching wiffle ball |
|  | Watching vehicle touring/racing | watching a stock car race, a drag race, a motocross race, snowmobiling, 4-wheeling |
|  | Watching volleyball | watching Wally ball |
|  | Watching walking | watching a walking sporting event |
|  | Watching water sports | watching a swim/dive meet, a water polo match, water aerobics |
|  | Watching weightlifting/strength training | watching weightlifting competition |
|  | Watching people working out, unspecified |  |
|  | Watching wrestling |  |
|  | Attending sporting events not elsewhere classified | watching discus, hang-gliding, ballooning, skydiving, windsurfing, bungee jumping, baton twirling, cheerleading, ping-pong, croquet game, archery, skeet-shooting, dart playing, boxing, javelin, pole vaulting |
| Religious/spiritual activities | Attending religious services | attending church services/synagogue/temple/mosque, attending funerals/attending wakes, memorial services, attending/hearing confession, attending a bar mitzvah/bat mitzvah/briss, attending a baptism/christening, leading blessings, attending/participating in weddings, attending church revival, attending a blessing, attending confirmation service |
|  | Participation in religious practices | reading the Bible/Koran/Torah/Talmud/scriptures, studying the Bible/Koran/sciptures, ushering for religious services, singing in the church choir, visiting graves/putting flowers on graves, rehearsing/decorating for religious ceremonies, attending a wedding rehearsal, cleaning up after religious ceremonies, attending church choir practice, attending a religious retreat, praying with others, preparing food for a Seder, praying with hh child, listening to Bible audio, lighting advent wreath, praying alone, saying prayers for a Seder, leading church choir, musical groups, playing the church organ, participating in church musical performance, opening advent calendar day, conducting religious rites in the home, meditating for religious purposes, preparing the sacrament/communion, lighting menorah candles (Hanukkah), attending Passover Seder, praying with nonhh child, attending church circle meeting |
|  | Religious education activities | attending confirmation class/attending Sunday school, attending Bible study, teaching/leading Bible study, distributing religious literature, reading (other: confirmation class), memorizing verses for confirmation class, teaching Sunday school, preparing materials for Bible study, teaching bat/bar mitvah class, attending pre-Cana class, leading religious youth group, reading/studying scriptures for Sunday school |
|  | Television (religious) | watching religious broadcasting |

| Volunteer-ing | Computer use (volunteer) | writing/sending e-mail, checking e-mail, designing website for volunteer org, loading computer software, computer use, unspecified, surfing the internet, setting up computer *– all (volunteer)* |
| --- | --- | --- |
|  | Organizing and preparing (volunteer) | preparing for a meeting, organizing volunteer materials, stuffing envelopes, completing paperwork, reviewing applications, filing, preparing/organizing schedules, addressing envelopes, re-shelving library books *- all (volunteer)* |
|  | Reading (volunteer) | reviewing notes, briefs, papers, reading books, journals, newspapers, reading the Bible, reading scriptures *– all (volunteer)* |
|  | Telephone calls (except hotline counseling) (volunteer) | making phone calls, answering phones *– all (volunteer)* |
|  | Writing (volunteer) | editing newsletters, writing letters/memos *- all (volunteer)* |
|  | Fundraising (volunteer) | collecting monetary donations, raising money for charitable causes, raising money for political candidates, selling items at a yard sale, soliciting pledges, selling tickets, planning a benefit, donating money, ringing a bell for the Salvation Army, selling non-food items at a concession stand, helping with a yard sale, selling items at a bazaar, selling raffle tickets, setting up a silent auction, selling Girl Scout cookies, running a race to raise money *– all (volunteer)* |
|  | Administrative & support activities not elsewhere classified (volunteer) | counted church offering, collecting signatures for a petition *- all (volunteer)* |
|  | Food preparation, presentation, clean-up (volunteer) | working in a soup kitchen, preparing food for a fundraiser, delivering/serving meals to shut-ins, baking cookies for the PTA bake sale, serving food to racers, distributing groceries at food bank, serving food at a homeless shelter, selling food or unknown items at a concession stand, donating food to a food bank *– all (volunteer)* |
|  | Collecting & delivering clothing & other goods (volunteer) | collecting clothing for disaster relief, distributing blankets at a homeless shelter, donating books, assembling gift baskets, donating tooys (voluteer), collecting toys, collecting used electronics, donating clothing, sorting books, sorting clothing, loading items into vehicle, unloading items from vehicle, collecting school supplies *– all (volunteer)* |
|  | Providing care (volunteer) | visiting shut-ins or the elderly, reading to the blind, acting as an interpreter, visiting with others, supervising children, spending time with hospice patients, walking dogs at animal shelter, playing with children, chaperoning *– all (volunteer)* |
|  | Teaching, leading, counseling, mentoring (volunteer) | being a Big Brother/Big Sister, tutoring, working a hotline for victim support svcs., coaching teams, assisting in a classroom, leading religious youth group, teaching Sunday school, teaching parenting classes, doing vocational training, leading a tour, teaching a class, leading story time at library, counseling at a halfway house, leading Boy Scout meeting, teaching/leading Bible study, teaching bat/bar mitzvah class, teaching/leading religious class, teaching confirmation class, teaching religious class, leading a blessing, teaching a cooking class, leading a choir, leading a musical group, providing a demo *- all (volunteer)* |
|  | Social service & care activities not elsewhere classified (volunteer) | sewing items to donate, making quilts for soldiers, knitting, making crafts *– all (volunteer)* |
|  | Building houses, wildlife sites, & other structures (volunteer) | building houses for Habitat for Humanity, building a wildlife refuge area, building playgrounds *– all (volunteer)* |
|  | Indoor & outdoor maintenance, repair, & clean-up (volunteer) | cleaning parks/streets, picking up trash, renovating a house, repairing tools or equipment, gardening, weeding, planting flowers, mowing the lawn, cleaning up yards, buildings, doing environmental clean-up, repairing cars, maintaining hiking trails, planting trees, watering trees, clearing brush, landscaping, painting *– all (volunteer)* |
|  | Indoor & outdoor maintenance, building & clean-up activities not elsewhere classified (volunteer) |  |
|  | Attending meetings, conferences, & training (volunteer) | attending training, attending meetings, attending conferences, attending seminars, serving on a board, serving on a finance committee, chairing a committee *– all (volunteer)* |
|  | Attending meetings, conferences, & training not elsewhere classified (volunteer) |  |
|  | Public health activities (volunteer) | donating blood, providing medical care *– all (volunteer)* |
|  | Public safety activities (volunteer) | being on duty as firefighter, patrolling for neighborhood crime watch *– all (volunteer)* |
|  | Public health & safety activities not elsewhere classified (volunteer) |  |

# Table S2. Characteristics of and rates of engagement in health-enhancing behaviours for all participants included in the Wellbeing Module compared to those included in the analytical sample.

| **Characteristic** | **Wellbeing Module  sample** (n=41,467) | **Health behaviour**  **sample** (n=11,144) |
| --- | --- | --- |
|  | **Mean (SD)** | |
| Age | 45.28 (18.66) | 46.99 (20.08) |
| Household size | 2.99 (1.58) | 2.91 (1.58) |
| Life satisfaction | 7.18 (1.99) | 7.18 (1.87) |
|  | **Proportion** | |
| Female | 52% | 53% |
| Race |  |  |
| White | 81% | 81% |
| Black | 12% | 12% |
| Asian | 4% | 5% |
| Other | 2% | 2% |
| Hispanic | 16% | 13% |
| Marital status |  |  |
| Married | 52% | 51% |
| Widowed/divorced/separated | 17% | 17% |
| Never married | 31% | 31% |
| Child under 18 in household | 39% | 35% |
| Metropolitan area | 85% | 85% |
| Region |  |  |
| Northeast | 18% | 18% |
| Midwest | 23% | 23% |
| Central | 36% | 36% |
| West | 22% | 23% |
| Education |  |  |
| High school or less | 45% | 43% |
| College | 25% | 23% |
| Undergraduate | 19% | 21% |
| Postgraduate | 11% | 13% |
| Employment status |  |  |
| Employed | 61% | 54% |
| Unemployed | 6% | 6% |
| Not in labour force | 17% | 19% |
| Retired | 16% | 22% |
| Annual family income |  |  |
| Less than $30,000 | 25% | 25% |
| $30,000 - $59,999 | 28% | 27% |
| $60,000 - $99,999 | 24% | 24% |
| $100,000 and over | 23% | 25% |
| Provided eldercare | 3% | 4% |
| Self-rated health |  |  |
| Excellent | 18% | 19% |
| Very good | 34% | 35% |
| Good | 31% | 30% |
| Fair | 13% | 12% |
| Poor | 4% | 4% |
| Disability that prevents work | 4% | 5% |
| High blood pressure | 29% | 31% |
| Well-rested |  |  |
| Very | 40% | 43% |
| Somewhat | 39% | 38% |
| A little | 15% | 14% |
| Not at all | 6% | 5% |
| Took pain medication | 28% | 28% |
| Feelings typical yesterday |  |  |
| Better | 26% | 29% |
| The same | 63% | 61% |
| Worse | 11% | 10% |
| Survey year |  |  |
| 2010 | 25% | 25% |
| 2012 | 26% | 26% |
| 2013 | 26% | 26% |
| 2021 | 23% | 23% |
| Behaviour engagement rates |  |  |
| Physical activity | 19% | 20% |
| Sports events | 1% | 1% |
| Participatory arts | 2% | 3% |
| Receptive arts | 3% | 3% |
| Reading | 22% | 20% |
| Social engagement | 41% | 47% |
| Religious/spiritual activities | 9% | 9% |
| Volunteering | 4% | 4% |

*Note.* Missing data on some variables in Wellbeing Module sample: n=12,991 missing on life satisfaction, n=325 metropolitan status, n=3457 family income, n=16 eldercare provision, n=16 disability status, n=148 self-rated health, n=244 high blood pressure, n=230 well-rested, n=74 pain medication, and n=12,965 feelings typical. Descriptives for health-enhancing behaviour sample from 40 multiply imputed datasets. All statistics weighted using ATUS individual-level weights.

# Table S3. Missingness in the health-enhancing behaviour sample (n=11,144).

|  | **Proportion missing** |
| --- | --- |
| Health-enhancing behaviours | - |
| Interacting with others | 0.2% |
| Activity location | 0.8% |
| Activity meaningful | 0.5% |
| Affect ratings |  |
| Happy | 0.5% |
| Sad | 0.2% |
| Pain | 0.1% |
| Stress | 0.1% |
| Tired | 0.2% |
| Gender | - |
| Age | - |
| Race | - |
| Ethnicity | - |
| Marital status | - |
| Household size | - |
| Child under 18 in household | - |
| Metropolitan area | 0.9% |
| Region | - |
| Education | - |
| Employment status | - |
| Annual family income | 8% |
| Provided eldercare | 0.1% |
| Self-rated health | 0.3% |
| Disability that prevents work | <0.1% |
| High blood pressure | 0.5% |
| Well-rested | 0.6% |
| Pain medication | 0.1% |
| Life satisfaction | 31% |
| Feelings typical yesterday | 31% |

# Table S4. Main analysis, before adjustment for covariates.

|  | **Happy** | | | **Sad** | | | **Stress** | | | **Tired** | | | **Pain** | | |
| --- | --- | --- | --- | --- | --- | --- | --- | --- | --- | --- | --- | --- | --- | --- | --- |
|  | Coef | 95% CI | p | Coef | 95% CI | p | Coef | 95% CI | p | Coef | 95% CI | p | Coef | 95% CI | p |
| Physical activity | - | - | - | - | - | - | - | - | - | - | - | - | - | - | - |
| Participatory arts | 0.16 | -0.07, 0.39 | 0.182 | 0.25 | -0.12, 0.62 | 0.183 | 0.26 | -0.01, 0.53 | 0.063 | -0.45 | -0.92, 0.01 | 0.058 | **-0.48** | **-0.88, -0.07** | **0.020** |
| Receptive arts | 0.15 | -0.06, 0.35 | 0.165 | -0.02 | -0.23, 0.18 | 0.815 | -0.06 | -0.28, 0.15 | 0.561 | -0.01 | -0.41, 0.39 | 0.974 | **-0.29** | **-0.56, -0.02** | **0.035** |
| Reading | **-0.41** | **-0.54, -0.27** | **<0.001** | **0.16** | **0.05, 0.28** | **0.004** | -0.06 | -0.23, 0.11 | 0.468 | 0.08 | -0.12, 0.28 | 0.438 | **-0.40** | **-0.53, -0.27** | **<0.001** |
| Social engagement | 0.10 | -0.02, 0.22 | 0.101 | **0.21** | **0.12, 0.31** | **<0.001** | 0.11 | -0.01, 0.22 | 0.067 | -0.08 | -0.26, 0.09 | 0.332 | **-0.48** | **-0.63, -0.34** | **<0.001** |
| Sports events | 0.04 | -0.30, 0.37 | 0.834 | 0.23 | -0.15, 0.61 | 0.228 | 0.01 | -0.35, 0.37 | 0.970 | -0.23 | -0.97, 0.52 | 0.548 | **-0.61** | **-0.92, -0.31** | **<0.001** |
| Religious/spiritual activities | **0.16** | **0.02, 0.30** | **0.028** | **0.18** | **0.07, 0.30** | **0.002** | -0.09 | -0.23, 0.05 | 0.189 | **-0.53** | **-0.75, -0.31** | **<0.001** | **-0.40** | **-0.58, -0.23** | **<0.001** |
| Volunteering | -0.23 | -0.47, 0.00 | 0.051 | 0.12 | -0.03, 0.27 | 0.128 | **0.48** | **0.27, 0.68** | **<0.001** | **-0.35** | **-0.68, -0.02** | **0.038** | **-0.41** | **-0.62, -0.20** | **<0.001** |

# Table S5. Interaction terms for effect modification by social context (interacting with others vs not interacting).

|  | **Happy** | | | **Sad** | | | **Stress** | | | **Tired** | | | **Pain** | | |
| --- | --- | --- | --- | --- | --- | --- | --- | --- | --- | --- | --- | --- | --- | --- | --- |
|  | Coef | 95% CI | p | Coef | 95% CI | p | Coef | 95% CI | p | Coef | 95% CI | p | Coef | 95% CI | p |
| Physical activity | - | - | - | - | - | - | - | - | - | - | - | - | - | - | - |
| Participatory arts | **-0.48** | **-0.92, -0.04** | **0.034** | -0.22 | -0.90, 0.45 | 0.517 | 0.13 | -0.37, 0.64 | 0.608 | 0.62 | -0.21, 1.45 | 0.145 | -0.42 | -1.09, 0.26 | 0.227 |
| Receptive arts | 0.08 | -0.29, 0.46 | 0.661 | -0.10 | -0.47, 0.27 | 0.595 | -0.09 | -0.50, 0.32 | 0.684 | 0.12 | -0.56, 0.79 | 0.733 | -0.06 | -0.49, 0.38 | 0.802 |
| Reading | 0.01 | -0.26, 0.27 | 0.966 | 0.08 | -0.12, 0.28 | 0.429 | 0.18 | -0.17, 0.52 | 0.312 | 0.35 | -0.04, 0.73 | 0.079 | 0.20 | -0.07, 0.46 | 0.145 |
| Social engagement | -0.02 | -0.29, 0.25 | 0.883 | -0.17 | -0.41, 0.07 | 0.176 | 0.00 | -0.27, 0.27 | 0.984 | 0.15 | -0.28, 0.59 | 0.493 | 0.31 | -0.03, 0.65 | 0.078 |
| Sports events | 0.25 | -0.66, 1.16 | 0.587 | 0.05 | -0.54, 0.65 | 0.860 | 0.31 | -0.28, 0.90 | 0.309 | 0.95 | -0.01, 1.91 | 0.053 | -0.29 | -1.23, 0.66 | 0.556 |
| Religious/spiritual activities | -0.26 | -0.54, 0.01 | 0.057 | 0.01 | -0.20, 0.22 | 0.936 | 0.24 | -0.01, 0.49 | 0.057 | 0.14 | -0.20, 0.48 | 0.427 | 0.17 | -0.12, 0.46 | 0.252 |
| Volunteering | -0.05 | -0.58, 0.48 | 0.858 | -0.08 | -0.36, 0.20 | 0.567 | -0.14 | -0.63, 0.35 | 0.587 | 0.27 | -0.43, 0.97 | 0.450 | -0.10 | -0.59, 0.39 | 0.683 |

# Table S6. Interaction terms for effect modification by location (outside home vs at home).

|  | **Happy** | | | **Sad** | | | **Stress** | | | **Tired** | | | **Pain** | | |
| --- | --- | --- | --- | --- | --- | --- | --- | --- | --- | --- | --- | --- | --- | --- | --- |
|  | Coef | 95% CI | p | Coef | 95% CI | p | Coef | 95% CI | p | Coef | 95% CI | p | Coef | 95% CI | p |
| Physical activity | - | - | - | - | - | - | - | - | - | - | - | - | - | - | - |
| Participatory arts | -0.10 | -0.57, 0.37 | 0.670 | -0.12 | -0.67, 0.43 | 0.674 | 0.20 | -0.25, 0.65 | 0.376 | **0.93** | **0.05, 1.82** | **0.039** | -0.30 | -0.77, 0.18 | 0.225 |
| Receptive arts | -0.17 | -0.59, 0.25 | 0.423 | 0.05 | -0.29, 0.39 | 0.782 | 0.18 | -0.22, 0.59 | 0.378 | 0.51 | -0.23, 1.26 | 0.176 | -0.18 | -0.66, 0.29 | 0.449 |
| Reading | **-0.42** | **-0.78, -0.07** | **0.020** | -0.19 | -0.57, 0.19 | 0.328 | 0.22 | -0.12, 0.57 | 0.198 | **-0.52** | **-0.95, -0.08** | **0.020** | -0.22 | -0.49, 0.05 | 0.104 |
| Social engagement | **-0.22** | **-0.42, -0.02** | **0.032** | 0.14 | -0.03, 0.31 | 0.101 | 0.12 | -0.11, 0.35 | 0.309 | 0.03 | -0.38, 0.44 | 0.888 | -0.10 | -0.36, 0.15 | 0.430 |
| Sports events | -0.06 | -2.02, 1.91 | 0.955 | -0.54 | -2.44, 1.35 | 0.573 | **0.77** | **0.04, 1.50** | **0.039** | -1.27 | -2.97, 0.42 | 0.142 | 0.38 | -0.07, 0.83 | 0.095 |
| Religious/spiritual activities | **-0.37** | **-0.65, -0.09** | **0.010** | 0.13 | -0.10, 0.36 | 0.251 | 0.12 | -0.14, 0.39 | 0.353 | -0.33 | -0.84, 0.17 | 0.198 | **-0.33** | **-0.63, -0.03** | **0.033** |
| Volunteering | **0.36** | **-0.08, 0.81** | **0.111** | 0.03 | -0.25, 0.31 | 0.845 | **-0.55** | **-0.92, -0.19** | **0.003** | -0.40 | -1.01, 0.20 | 0.193 | **-0.39** | **-0.75, -0.02** | **0.037** |

# Table S7. Interaction terms for effect modification by activity meaning (very meaningful vs less meaningful).

|  | **Happy** | | | **Sad** | | | **Stress** | | | **Tired** | | | **Pain** | | |
| --- | --- | --- | --- | --- | --- | --- | --- | --- | --- | --- | --- | --- | --- | --- | --- |
|  | Coef | 95% CI | p | Coef | 95% CI | p | Coef | 95% CI | p | Coef | 95% CI | p | Coef | 95% CI | p |
| Physical activity | - | - | - | - | - | - | - | - | - | - | - | - | - | - | - |
| Participatory arts | -0.11 | -0.53, 0.32 | 0.629 | -0.37 | -1.02, 0.28 | 0.260 | -0.44 | -0.95, 0.06 | 0.086 | -0.73 | -1.53, 0.07 | 0.076 | -0.22 | -0.93, 0.48 | 0.534 |
| Receptive arts | 0.20 | -0.15, 0.55 | 0.258 | -0.28 | -0.65, 0.10 | 0.150 | -0.02 | -0.44, 0.40 | 0.929 | -0.38 | -1.11, 0.34 | 0.302 | -0.10 | -0.58, 0.38 | 0.683 |
| Reading | 0.17 | -0.05, 0.40 | 0.131 | -0.04 | -0.29, 0.20 | 0.723 | -0.02 | -0.27, 0.23 | 0.889 | -0.30 | -0.63, 0.04 | 0.084 | 0.03 | -0.23, 0.29 | 0.814 |
| Social engagement | 0.19 | -0.01, 0.40 | 0.067 | 0.12 | -0.07, 0.30 | 0.229 | **-0.26** | **-0.49, -0.03** | **0.024** | -0.28 | -0.59, 0.04 | 0.085 | -0.04 | -0.30, 0.23 | 0.798 |
| Sports events | 0.39 | -0.07, 0.84 | 0.099 | -0.15 | -0.70, 0.39 | 0.580 | 0.34 | -0.34, 1.02 | 0.325 | -0.94 | -2.06, 0.18 | 0.099 | -0.04 | -0.50, 0.42 | 0.871 |
| Religious/spiritual activities | **0.39** | **0.12, 0.66** | **0.005** | -0.11 | -0.35, 0.12 | 0.350 | **-0.44** | **-0.74, -0.13** | **0.005** | **-0.63** | **-1.03, -0.23** | **0.002** | -0.12 | -0.53, 0.29 | 0.552 |
| Volunteering | 0.31 | -0.10, 0.72 | 0.136 | 0.03 | -0.26, 0.31 | 0.851 | **-0.76** | **-1.25, -0.27** | **0.002** | -0.58 | -1.23, 0.07 | 0.078 | -0.11 | -0.48, 0.26 | 0.571 |

# Table S8. Main analysis, adjusted for covariates, limited to the complete case sample (7,382 individuals, 8,558 observations).

|  | **Happy** | | | **Sad** | | | **Stress** | | | **Tired** | | | **Pain** | | |
| --- | --- | --- | --- | --- | --- | --- | --- | --- | --- | --- | --- | --- | --- | --- | --- |
|  | Coef | 95% CI | p | Coef | 95% CI | p | Coef | 95% CI | p | Coef | 95% CI | p | Coef | 95% CI | p |
| Physical activity | - | - | - | - | - | - | - | - | - | - | - | - | - | - | - |
| Participatory arts | 0.12 | -0.12, 0.36 | 0.341 | 0.09 | -0.40, 0.57 | 0.726 | 0.09 | -0.20, 0.39 | 0.537 | -0.60 | -1.24, 0.03 | 0.062 | -0.50 | -1.00, 0.00 | 0.051 |
| Receptive arts | 0.17 | -0.06, 0.41 | 0.145 | -0.07 | -0.30, 0.17 | 0.579 | **-0.24** | **-0.49, -0.00** | **0.049** | -0.43 | -0.86, 0.00 | 0.052 | **-0.38** | **-0.70, -0.06** | **0.019** |
| Reading | **-0.23** | **-0.43, -0.04** | **0.019** | 0.00 | -0.13, 0.14 | 0.953 | **-0.21** | **-0.41, -0.01** | **0.043** | -0.07 | -0.35, 0.22 | 0.654 | **-0.49** | **-0.65, -0.32** | **<0.001** |
| Social engagement | 0.02 | -0.15, 0.20 | 0.810 | 0.09 | -0.02, 0.21 | 0.092 | 0.04 | -0.10, 0.19 | 0.567 | -0.25 | -0.53, 0.02 | 0.066 | **-0.48** | **-0.64, -0.32** | **<0.001** |
| Sports events | -0.12 | -0.51, 0.27 | 0.536 | 0.32 | -0.19, 0.83 | 0.219 | -0.06 | -0.50, 0.38 | 0.778 | -0.21 | -1.23, 0.81 | 0.689 | **-0.55** | **-0.87, -0.23** | **0.001** |
| Religious/spiritual activities | 0.02 | -0.17, 0.21 | 0.839 | 0.04 | -0.10, 0.18 | 0.585 | -0.10 | -0.28, 0.07 | 0.258 | **-0.53** | **-0.83, -0.22** | **0.001** | **-0.44** | **-0.65, -0.22** | **<0.001** |
| Volunteering | **-0.33** | **-0.64, -0.02** | **0.038** | 0.13 | -0.05, 0.32 | 0.149 | **0.50** | **0.24, 0.76** | **<0.001** | -0.38 | -0.77, 0.00 | 0.052 | **-0.46** | **-0.70, -0.21** | **<0.001** |

# *Figure S1. Associations between health-enhancing behaviours (versus physical activity) and happiness, stratified by social context. Activities done when not interacting compared to physical activity when not interacting, and activities done when interacting compared to physical activity when interacting.*

# *Figure S2. Associations between health-enhancing behaviours (versus physical activity) and happiness, stratified by location. Activities done at home compared to physical activity at home, and activities done outside home compared to physical activity outside home.*

# *Figure S3. Associations between health-enhancing behaviours (versus physical activity) and happiness, stratified by activity meaningfulness. Less meaningful activities compared to less meaningful physical activity, and very meaningful activities compared to very meaningful physical activity.*

# *Figure S4. Main analysis with social engagement as reference category.*

# *Figure S5. Main analysis with reading as reference category.*

# Analysis of net affect

Net affect was calculated as happiness minus sadness, meaning scores ranged from -6 to 6. A low score indicates more sadness than happiness, a score around 0 indicates similar levels of happiness and sadness (either both high or both low), and a high score indicates more happiness than sadness.

On average, scores were high, reflecting higher ratings of happiness than sadness across all health-enhancing behaviours. Descriptively, net affect was highest for receptive arts (mean=4.69, SE=0.14), followed by physical activity (mean=4.61, SE=0.06), and sports events (mean=4.58, SE=0.21). In contrast, net affect was lowest for reading (mean=3.77, SE=0.08) and participatory arts (mean=3.96, SE=0.29).

We then used multilevel linear regression models to test whether net affect differed across the eight health-enhancing behaviours after adjusting for confounders (Figure S6). Compared to physical activity, there was strong evidence only that net affect was lower for reading and volunteering. This indicates that the difference between happiness and sadness was smaller for reading and volunteering than for physical activity. Net affect also trended towards being lower for social engagement than physical activity.

# *Figure S6. Main analysis using net affect (happiness – sadness) as the outcome.*
